# Use of *Limonium vulgare* Mill. and Stevia in Rice Pudding Production and Determination of Quality Parameters During Storage

**DOI:** 10.1002/fsn3.70297

**Published:** 2025-05-20

**Authors:** Fadime Seyrekoğlu, Sultan Acun, Aslı Yildirim Vardin, Ali Göncü

**Affiliations:** ^1^ Food Processing Department, Suluova Vocational School Amasya University Amasya Türkiye; ^2^ Food Engineering Department, Engineering Faculty Aydın Adnan Menderes University Aydın Türkiye; ^3^ Food Processing Department, Çine Vocational School Aydın Adnan Menderes University Aydın Türkiye

**Keywords:** HMF, *Limonium vulgare* Mill., rice pudding, stevia

## Abstract

In recent times, the increasing prevalence of health problems such as obesity, hypertension, and diabetes has led consumers to seek healthier food options. There has been a growing trend toward natural alternative foods for healthy eating. People are increasingly turning to low‐calorie functional foods. In this study, stevia, an alternative to sugar, and *Limonium vulgare* Mill., which is particularly rich in phenolic compounds and has high antioxidant activity, were used in the production of rice pudding (a Turkish milk pudding) with the aim of reducing sugar usage. Thus, a new functional dessert product was produced. As a result, rice pudding samples were produced with the addition of stevia, sugar, *Limonium vulgare* Mill. extract at determined ratios (5%, 10%, and 15%), and sugar with *Limonium vulgare* Mill. liquid extract at determined ratios (5%, 10%, and 15%). Some quality parameters of the obtained rice pudding samples were examined. The dry matter values of the samples were found to be in the range of 21.20–25.63, L* values 74.41–83.32, a* values 2.20–7.14, b* values 18.34–21.92, pH values 6.96–7.56, and water holding capacity values 29.73–74.08. When the overall liking scores were examined, the sample containing stevia +10% *Limonium vulgare* Mill. extract received the highest score on the first day of storage, while the sample containing stevia +15% *Limonium vulgare* Mill. extract received the highest score on the last day of storage. The total phenolic content increased with the increasing amount of added extract. The %DPPH inhibition increased proportionally with both the increasing amount of added extract and the storage time. In terms of microbiology, the yeast and mold count was at its maximum level in the sample containing stevia +15% *Limonium vulgare* Mill. extract, while no total coliform group microorganisms were found in any of the samples. The major volatile compounds identified in the sütlaç samples were benzene, 1,3‐dimethylbenzene, p‐xylene, o‐xylene, γ‐terpinene, p‐cymene, 2‐nonanone oxime, methoxyphenyl, vanillin, and 18,18′‐bi‐1,4,7,10,13,16‐hexaoxacyclononadecane. Samples containing sugar combined with 5%–15% *Limonium vulgare* Mill. extract, as well as those containing stevia and 15% *Limonium vulgare* Mill. extract, exhibited HMF (hydroxymethylfurfural). Conversely, no hydroxymethylfurfural was detected in samples containing both sugar and stevia, or in those containing only *Limonium vulgare* Mill. extract at different concentrations. Our results indicate that substituting sugar with stevia in rice pudding formulations may mitigate hydroxymethylfurfural accumulation in specific formulations. With this study, the contributions of stevia and 10%–15% *Limonium vulgare* Mill. extract to the model food were investigated, and the usable ratios were determined. As a result of the analyses, the usability and acceptability of stevia and *Limonium vulgare* Mill. extract were determined, and their use in foods will be widespread.

## Graphical abstract

When the literature is examined, there are very few studies on milk desserts, especially rice pudding. In this study, the use of *Limonium vulgare* Mill. plant and stevia, which has never been used in rice pudding, was discussed. A functional milk dessert production was presented to the literature by comparing the use of stevia and sugar. The study is original with the materials used and comparison. In addition, physicochemical, microbiological, sensory, flavor, and HMF analyses were performed on the obtained rice pudding, and many properties of the products obtained were investigated and discussed in detail. By reducing the use of sugar, it is aimed to prevent many health problems and to popularize the use of rice pudding, which people love to consume. Especially for children, diabetics, and people with obesity problems, a dessert that can be consumed with health and pleasure has been produced.

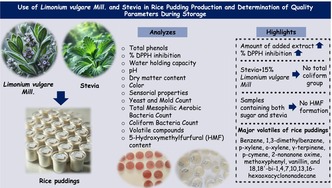

## 1 Introduction

The interest in plant‐based natural products in the food and pharmaceutical industries is particularly noteworthy, especially in terms of food safety and functional food ingredients. Bioactive plant extracts with high antioxidant properties prevent oxidative deterioration in foods, extending shelf life and reducing the formation of toxic compounds, thus preserving nutritional value. These bioactive molecules found in functional foods and dietary supplements have the potential to reduce the risk of cardiometabolic and tumoral diseases and provide a natural contribution to health. With these properties, plant‐based products both enhance food quality and contribute to healthy eating trends (Medini et al. 2014; Panta et al. 2014; Souid et al. 2019).

*Limonium vulgare* Mill., commonly referred to as sea lavender, is a halophytic species exhibiting remarkable resilience to adverse environmental conditions. Its adaptation to such stressors has resulted in alterations in its endogenous chemical composition. Notably, this adaptation process seems to enhance the biosynthesis of bioactive compounds within the plant. Increased levels of phenolic compounds, known as secondary metabolites, in extracts from *Limonium vulgare* Mill. have been shown to enhance antioxidant, antimicrobial, and antiviral properties, thus promoting human health (Lellau and Liebezeit 2003; Souid et al. 2019; Degirmenci and Erkurt 2020; Carius et al. 2022).

Added sugars are widely used in foods not only as a source of carbohydrates but also to enhance flavor, texture, color, and shelf life (Clemens et al. 2016). Sugar types such as sucrose and high‐fructose corn syrup (HFCS) are commonly used as sweeteners in many processed foods, highlighting the versatile use of sugar in the food industry. The presence of added sugars in sweetened beverages, packaged snacks, and other processed foods is significant in terms of the nutritional value and functionality of these foods. However, it has been suggested that sugar, especially with its high‐fructose content, can directly increase the risk of metabolic diseases by disrupting lipid and carbohydrate metabolism. Excessive consumption of added sugars can lead to a positive energy balance, contributing to weight gain and adiposity. This, in turn, increases the risk of metabolic disorders such as type 2 diabetes and cardiovascular disease. While the link between sugar and dental caries is well established, the precise role of sugar in the etiology of these metabolic conditions remains a subject of ongoing debate (Clemens et al. 2016; Johnson et al. 2009; Walton et al. 2023). Given these concerns, stevia has emerged as a popular low‐calorie sweetener alternative.

*Stevia rebaudiana* Bertoni, a perennial shrub belonging to the Compositae family and native to South America, is now widely cultivated in Asia, Europe, and North America. Unique in its sweetness, *Stevia rebaudiana* provides 100–300 times the sweetness of sucrose because of its steviol glycosides (Lemus‐Mondaca et al. 2012). *Stevia rebaudiana* , a natural sweetener, is a rich source of various bioactive compounds including vitamins, minerals, amino acids, flavonoids, and phenolic compounds, contributing to its diverse health benefits (Wölwer‐Rieck 2012; Ahmad et al. 2020). Owing to its increasing utilization as a sugar substitute in the food and pharmaceutical sectors, stevia has been extensively studied for its pharmacological properties, demonstrating significant antidiabetic, anti‐obesity, antioxidant, antitumor, antihypertensive, and antimicrobial effects. Numerous studies have consistently demonstrated the absence of teratogenic, carcinogenic, and mutagenic effects associated with stevia leaf consumption, thereby establishing its safety profile (Abbas Momtazi‐Borojeni et al. 2017). Owing to its functional properties and pleasant taste as a natural sweetener, stevia has garnered significant attention in the health food industry and is expected to gain importance as a sugar substitute in the future (Ahmad et al. 2020).

Rice pudding is a culinary preparation involving the slow cooking of short‐grain rice in milk. The starch granules within the rice undergo gelatinization during the cooking process, resulting in a viscous, creamy texture with distinct rice grains. Sweeteners and spices are added to impart flavor. This dessert, known as “rice pudding” in Turkish, “Milchreis” in Germany, and “Kheer” in India, is a globally popular dish with diverse regional variations (Sattar et al. 2017; Güldemir et al. 2018; Sompolski and Hefft 2022). Commercial rice puddings are typically distributed in sealed plastic containers under refrigeration and may be formulated as ready‐to‐mix products. Their high water activity (up to 0.75) and slightly acidic pH (approximately 6.5) render them susceptible to microbial spoilage, necessitating cold chain storage below 4°C (Papageorgiou et al. 2003). While synthetic preservatives are commonly incorporated to extend shelf life, the growing consumer preference for minimally processed foods has driven interest in natural alternatives such as plant and spice extracts. However, the antimicrobial potential of these natural extracts in dairy desserts has not been fully elucidated, with most studies limited to in vitro evaluations (Degirmenci and Erkurt 2020).

Rice pudding, made with nutrient‐rich rice, has significant nutritional value. Inadequate nutrient intake during childhood has been linked to attention deficits, a nutritional gap that rice pudding can help bridge (Acharya et al. 2018); furthermore, by reducing its sugar content, it can be effectively incorporated into the dietary management of diabetes (Nicklas et al. 2014; Suttireung et al. 2019). The incorporation of antioxidants and antimicrobial agents during rice pudding production not only enriches its nutritional composition but also extends its shelf life by suppressing microbial proliferation (Papageorgiou et al. 2003).

The present study aimed to formulate functional rice pudding by utilizing the antioxidant and antimicrobial properties of *Limonium vulgare* Mill. extract and stevia, with the primary objective of enhancing the shelf life of nutrient‐rich rice puddings. To assess the quality and shelf life of the developed products, a comprehensive evaluation involving physical, chemical, sensory, and microbiological analyses was conducted.

The aim of this study is to produce functional rice pudding using *Limonium vulgare* Mill. extract and stevia. Rice pudding is not only a traditional dessert but also has a nutritious content; however, its short shelf life and high sugar content limit its appeal to those seeking healthier options and long‐lasting products. In this study, the *Limonium vulgare* Mill. extract, known for its natural antioxidant and antimicrobial properties, along with the low‐calorie natural sweetener stevia, has been incorporated into the rice pudding formulation. The main goal of this study is to offer consumers a healthier option by extending the shelf life of the rice puddings while preserving their nutritional value and reducing sugar content. The quality and shelf life of the developed products will be thoroughly evaluated through physical, chemical, sensory, and microbiological analyses.

## 2 Material and Methods

### 2.1 Materials

The *Limonium vulgare* Mill. plants were collected from the Yedikır Region of Suluova, Amasya in August 2023. The identification of the plant was performed at the Department of Biology, Amasya University (Prof. Dr. Duygu Kılıç). The milk, rice, sugar, starch, and rice flour used in the rice pudding production were bought from a local market in Suluova. Stevia powdered sugar was supplied by Fibrelle Company. All chemicals used in the analyses were 100% pure.

### 2.2 Extraction of *Limonium vulgare* Mill. Plants

In this study, an optimization process was carried out to determine the most suitable extraction conditions based on the parameters reported by Carius et al. (2022) and Safarzaei et al. (2020). The optimized conditions were applied for the extraction of *Limonium vulgare* Mill.

Ultrasound‐assisted extraction (UDSE) was employed to extract bioactive compounds from *Limonium vulgare* Mill. The plant‐to‐solvent ratio was set at 1:30, using a solvent mixture composed of 20% ethanol and 80% water. The extraction was performed at 70°C for 60 min using ultrasonic waves. Ethanol was removed from the obtained extract using a rotary evaporator. The UDSE method was found to be effective for the extraction of flavonoids from *Limonium vulgare* Mill.

### 2.3 Methods

#### 2.3.1 Production of Rice Pudding

The experiment was conducted with eight different rice pudding groups (rice pudding with sugar [control], rice pudding with stevia, rice pudding with sugar and 5% *Limonium vulgare* Mill. extract, rice pudding with sugar and 10% *Limonium vulgare* Mill. extract, rice pudding with sugar and 15% *Limonium vulgare* Mill. extract, rice pudding with stevia and 5% *Limonium vulgare* Mill. extract, rice pudding with stevia and 10% *Limonium vulgare* Mill. extract, and rice pudding with stevia and 15% *Limonium vulgare* Mill. extract), three different storage times (1st, 5th, and 10th days), and two replications that were carried out as. Throughout the storage study, the rice pudding samples were stored under controlled refrigerated conditions at 4°C ± 1°C. This temperature range was maintained consistently using a laboratory‐grade refrigerator to replicate typical cold storage conditions and ensure the stability of physicochemical and microbiological parameters during the evaluation period.

Ingredients and production of rice pudding were optimized with the preliminary studies. Rice pudding was produced under optimum conditions as a result of the sensory analysis.

**FIGURE 1:**
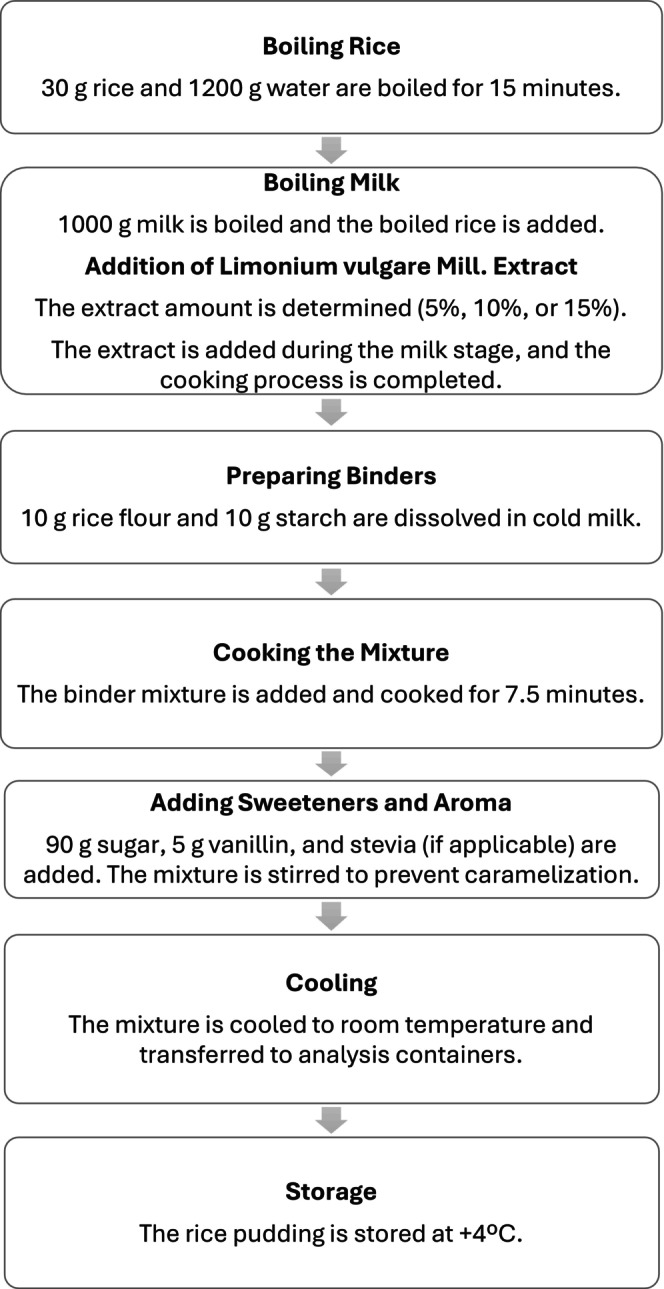
Flowchart of the rice pudding production process.

Rice (30 g) was boiled with 1200 g of water for 15 min (Figure 1). In another pot, 1000 g of milk was boiled, rice was added, and the heat treatment was continued. Take some of the mixture and cool it aside. Rice flour (10 g) and starch (10 g) were dissolved in cold milk. The mixture with the binders added to the pot was kept on the stove for 7.5 min. Sugar (90 g) and vanillin (5 g) were added to the mixture, and stirring was continued to prevent caramelization. After 15 min, the stove was turned off, and the rice pudding was allowed to come to room temperature. Then, the rice pudding was taken into analysis containers and stored at +4°C (Figure 1).

First, rice pudding with sugar was produced. Then, rice pudding containing the same amount of stevia was produced. In the rice pudding production containing *Limonium vulgare* Mill. extract, the amount of milk was reduced by 5%, 10%, and 15% by adding 5%, 10%, and 15% *Limonium vulgare* Mill. extract. *Limonium vulgare* Mill. extract was added during the milk addition stage, and the cooking process was completed. The rice pudding production process is illustrated in Figure 1.

### 2.4 Analysis

#### 2.4.1 Determination of Dry Matter Content

Dry matter analysis is a method used to determine the proportion of insoluble polysaccharides such as cellulose and starch in food. For this analysis, drying containers were kept in an oven at 100°C for an (1) hour and then cooled in a desiccator. Approximately 5 g of sample was weighed into the tared containers and spread in a thin layer. The samples were dried at 100°C for 1.5–2 h and cooled in a desiccator until the samples reached constant weight. Finally, the containers were weighed, and the % dry matter content was calculated (Metin 2012).

$$\mathrm{Dry}\;\text{matter}\;\left(\%\right)=\left(\text{G3}-\text{G1}\right)/\left(\text{G2}-\text{G1}\right)\times 100$$

where, G1: weight of empty drying container (g), G2: weight of container with sample (g), G3: weight of container with dried sample (g).

#### 2.4.2 Determination of pH

The pH of a sample solution was analyzed using a pH meter (Hanna Instruments HI 83141, Italy) standardized by immersing the probe in a 50‐mL beaker containing 25 mL of buffer solution. After cleaning and drying the probes, the pH of the sample solution was adjusted to 25°C, and the pH analysis was performed by immersing the probe of the device (Emirmustafaoğlu and Coşkun 2017).

#### 2.4.3 Determination of Colors

Color values were measured using a color determination device (Konica Minolta, CR400, Japan) with three parallel measurements. The values measured were CIE L* (brightness), a* (positive values indicate an increase in the values of red, negative values indicate an increase in the values of green), and b* (positive values indicate an increase in the values of yellow, negative values indicate an increase in the values of blue).

#### 2.4.4 Determination of Sensory Properties

Sensory evaluation of the prepared rice puddings was carried out using the form given in Annex 1 in terms of color‐appearance, structure‐consistency, taste‐odor, and overall acceptability (Metin 2012; Kadağan 2015). The sensory evaluation of the prepared rice puddings was conducted based on the criteria of color‐appearance, structure‐consistency, taste‐odor, and overall acceptability (Metin; Kadağan). Rice pudding samples were coded and presented to the panelists. Sensory analyses were carried out with a 10‐member panel group consisting of faculty members of Amasya University Suluova Vocational School on the 1st, 5th, and 10th days of storage.

#### 2.4.5 Determination of Water Holding Capacity

Water holding capacity was determined using the method described by Granato et al. (2010). In this method, 10 g samples from each sample were centrifuged at 20°C, 5000 rpm for 40 min (Sigma, 2‐16K, Germany). The serum phase formed after centrifugation was removed from the sample. The remaining sample mass was weighed, and the following formula was used to determine the water holding capacity:

$$\text{Water holding capacity}=\left(\mathrm{Ws}/\mathrm{Wi}\right)\times 100$$

Ws: pellet weight (g), Wi: sample weight (g).

#### 2.4.6 Determination of Total Phenolic Content and %DPPH Inhibition

##### 2.4.6.1 Preparation of Rice Pudding Extract

Ten grams of rice pudding were suspended in 20 mL of 99.5% ethanol (v/v) and mixed thoroughly until a smooth, fine slurry was formed. This ethanolic solution was then centrifuged at 3000 × *g* for 10 min. The supernatant, which contains the desired rice pudding extract, was collected. To prepare solutions for analysis of total phenol content and antioxidant capacity, aliquots of the extract were diluted to various concentrations (0.1, 0.2, 0.3, and 0.4 kg/L) using 75% ethanol (Sattar et al. 2017).

##### 2.4.6.2 Determination of Total Phenols

The extracted sample (20 μL) was diluted with water (1580 μL) for a total volume of 1600 μL. Then Folin–Ciocalteu reagent (100 μL) was added to the diluted sample. The mixture was thoroughly mixed and incubated for 8 min. After sodium carbonate (300 μL) solution was added to the mixture. The solution was incubated for 2 h at room temperature. The absorbance of the final solution was measured at 765 nm using a UV–visible spectrophotometer (V670; Jasco, Tokyo, Japan). A standard curve of gallic acid was used to quantify the amount of phenolics in the sample based on its absorbance. The results were expressed as milligrams of gallic acid equivalent per gram (following a method described by Waterhouse in 2002).

##### 2.4.6.3 Determination of % DPPH Inhibition

This procedure follows the method established by Marinova and Batchvarov (2011) to assess free radical scavenging activity. An ethanolic dilution of the sample (1.5 mL) was put in a test tube. DPPH solution (0.5 mL) was added to the sample in the test tube. The mixture was kept in darkness for 30 min to allow the sample to interact with the DPPH radicals. After incubation, the absorbance of the mixture was measured at 517 nm using a UV–visible spectrophotometer (V670; Jasco). A diluted blank solution is used for reference during the absorbance measurement. Calibration curves prepared with ascorbic acid were used to convert the measured absorbance value into an “ascorbic acid equivalent antioxidant capacity.” This allows comparison of the sample's free radical scavenging activity to a well‐known antioxidant (ascorbic acid).

#### 2.4.7 Determination of Microbiological Values

##### 2.4.7.1 Sample Preparation for Microbiological Analysis

Sterile Ringer's solution was used as the diluent for rice pudding samples. For each sample, 10 g of rice pudding was weighed into 90 mL of Ringer's solution and homogenized to prepare a 10^−1^ dilution. 10^−2^ dilution was prepared by transferring 1 mL of the 10^−1^ dilution to a tube containing 9 mL of Ringer's solution. Dilutions were prepared in this manner up to 10^−4^. Inoculations were made from the 10^−1^, 10^−2^, 10^−3^, and 10^−4^ dilutions for yeast, mold, coliforms, and total bacteria. Results were expressed as log CFU/g.

##### 2.4.7.2 Yeast and Mold Count

Yeast extract glucose chloramphenicol agar (Merck 1.16000) medium was used for yeast and mold counting. Inoculation was performed according to the pour plate method. The plates were incubated at 25°C for 3 days for yeasts and 5 days for molds. The colonies formed were counted (Ethiopian Standard Agency 2012).

##### 2.4.7.3 Total Mesophilic Aerobic Bacteria Count

Plate count agar (Merck 1.05463) medium was used for total bacterial count. Inoculation was performed according to the pour plate method. The plates were incubated at 37°C for 48 h, and the colonies formed were counted (Harrigan and Cance 1976).

##### 2.4.7.4 Coliform Bacteria Count

Violet Red Bile Agar (Merck 1.01406) medium was used for the enumeration of coliform bacteria. Inoculation was done by the pour plate method, followed by the addition of a second layer of VRB Agar. The plates were incubated at 37°C for 24 h, and the colonies formed were counted (Hitchins et al. 1992).

#### 2.4.8 Determination of Volatile Compounds

The volatile components of the samples were analyzed by gas chromatography–mass spectrometry (GC–MS) employing the solid‐phase micro‐extraction (SPME) technique (D'Auria et al. 2004). First, 2 g of the sample was weighed into vials with septum caps and incubated at 40°C for 30 min to facilitate the transfer of aroma compounds from the sample to the surrounding medium. A DVB/CAR/PDMS fiber (65 μm, Supelco, Bellefonte, PA, USA) was introduced into the headspace of the vial for SPME and maintained at 40°C for an additional 30 min. Subsequently, the fiber containing volatile components was transferred to the GC–MS sampling port. The analysis of volatile components was performed using a capillary column (Restek Rxi‐5 ms, USA; 30 m × 0.25 mm ID × 0.25 μm). Injection port temperature was 250°C, interface temperature was 250°C, while desorption time was 3 min. High‐purity helium was the mobile phase. Injection was done in splitless mode. GC‐column temperature was as follows: Starting with 1 min at 50°C, followed by a gradual increase of 3°C/min to 200°C, then 8°C/min to 250°C, with a hold at 250°C for 5 min; MS‐scan mode set to 35–450 m/z; ionization energy at 70 eV. Identification of volatile compounds was carried out using libraries registered on GC–MS.

#### 2.4.9 Determination of HMF Content

HMF content of the samples was determined by high‐performance liquid chromatography (HPLC, Agilent Technologies, California, USA). First, 6.9 g of sample was weighed and diluted to 50 mL with pure water. Then, the samples were filtered through a 0.45‐μm syringe tip membrane filter. The mobile phase consisted of 80% pure water and 20% methanol at a flow rate of 1 mL/min. Chromatographic separation was carried out on a DAD detector at a wavelength of 285 nm at room temperature. Separation was performed using a C18 column (150 × 4.6 mm with 3‐μm particle size) (Koç 2015). The amount of HMF in the samples was determined quantitatively using the calibration curve prepared with HMF standards at different concentrations.

#### 2.4.10 Determination of Statistical Values

Statistical analysis was performed using ANOVA for normally distributed groups to interpret the differences between the results of physicochemical, microbiological, and sensory analyses and the differences that occurred in the groups with storage. To determine which group is different from the other in the differences determined with ANOVA, “Tukey” tests were used if the main mass variances were the same (Özdamar 2009). The data were analyzed using the SPSS (Statistical Package for the Social Sciences) 18.00 package program.

## 3 Results and Discussion

The dry matter content values of the rice pudding samples are presented in Table 1. The effects of both storage and sample type were evaluated statistically. A decline in dry matter was observed across all samples as storage progressed. On the first day of storage, an increase in the proportion of *Limonium vulgare* Mill. extract led to an increase in the dry matter content. The dry matter from the extracts contributed to an increase in the total dry matter content. The maximum dry matter ratio was observed in the KS15 sample (containing 15% *Limonium vulgare* Mill. extract and sugar) on the first day of storage, while the minimum dry matter ratio was detected in the KS (control sugar) sample on the 10th day of storage. These findings indicate a consistent decrease in dry matter content over the storage period. The dry matter content of the optimized vegan rice pudding samples prepared using different plant‐based milks and evaluated for their rheological, textural, and sensory properties was found to range from 14.13 to 22.66 (Karimidastjerd et al. 2024). The dry matter content in our control samples was found to be tightly clustered between 21.20 and 22.34. Although the overall range for all samples was somewhat expanded (21.20–25.63), a comparison with previous findings on low‐fat and whole milk (21.06–22.66) suggests that our control group (lacking *Limonium vulgare* Mill. extract) exhibited a statistically similar dry matter content. The dry matter content of rice pudding samples in this study, which examined the impact of various hydrocolloid combinations and storage durations, ranged from 28.33% to 29.78% (Kadagan and Arslan 2022). These results are slightly higher compared to those reported by Köylü (2019) (23.95%–26.75%) for rice pudding and Djaoud et al. (2020) (21.52%–23.02%) for dairy desserts. Differences in dry matter values are a result of the varying compositions of raw materials, the addition of different ingredients during production, and the duration of storage.

**TABLE 1:** Physicochemical analyses of rice pudding samples.

Physicochemical analyses	Storage days	Samples							
		KS	KSE	KS5	KSE5	KS10	KSE10	KS15	KSE15
Dry matter (%)	First day	21.60^Ea^ ± 0.64	22.34^DEa^ ± 0.65	23.97^Ba^ ± 0.48	22.71^CDa^ ± 0.98	23.85^Ba^ ± 0.18	23.56^BCa^ ± 0.38	25.63^Aa^ ± 0.43	25.20^Aa^ ± 0.59
	Fifth day	21.77^Ba^ ± 1.96	21.63^Ba^ ± 0.24	21.92^Bb^ ± 0.34	22.47^Ba^ ± 0.19	22.80^Ba^ ± 0.58	22.49^Bc^ ± 0.13	24.14^Ab^ ± 0.14	22.47Bb ± 0.49
	Tenth day	21.20^Da^ ± 0.01	21.91^CDa^ ± 0.38	22.49^BCb^ ± 0.19	22.15^BCDa^ ± 0.69	22.77^BCa^ ± 1.19	23.03^Bb^ ± 0.12	23.99^Ab^ ± 0.25	22.86^BCb^ ± 0.51
L* (color anlayses)	First day	82.30^Aa^ ± 1.98	81.75^Aa^ ± 2.41	80.89^Aa^ ± 0.43	81.52^Aa^ ± 0.15	77.64^Ba^ ± 1.23	75.05^Cc^ ± 1.31	76.63^BCa^ ± 0.36	76.11^BCa^ ± 0.49
	Fifth day	80.71^Ab^ ± 0.93	82.47^Aa^ ± 0.24	79.77^BCa^ ± 0.65	81.40^ABa^ ± 0.34	78.17^Da^ ± 0.86	78.60^CDa^ ± 0.15	76.70^Ea^ ± 0.84	75.13^Fa^ ± 1.13
	Tenth day	83.32^Aa^ ± 1.87	82.88^ABa^ ± 1.12	81.02^BCa^ ± 0.95	80.11^Cb^ ± 0.16	74.41^Eb^ ± 1.01	76.65^Db^ ± 0.34	74.75^DEb^ ± 1.08	75.13^DEa^ ± 1.81
a* (color anlayses)	First day	3.08^Ga^ ± 0.39	3.53^Fb^ ± 0.27	4.14^Ea^ ± 0.33	4.68^Db^ ± 0.04	5.58^Ca^ ± 0.05	6.33^Ba^ ± 0.16	6.93^Aab^ ± 0.20	6.68^ABa^ ± 0.05
	Fifth day	3.18^Ea^ ± 0.22	3.92^Dab^ ± 0.23	4.34^CDa^ ± 0.30	4.97^Ca^ ± 0.12	5.80^Ba^ ± 0.16	6.23^Ba^ ± 0.03	7.15^Aa^ ± 0.15	6.23^Ba^ ± 1.02
	Tenth day	2.20^Db^ ± 0.16	4.15^Ca^ ± 0.31	4.45^Ca^ ± 0.23	4.80^Cb^ ± 0.02	4.66^Cb^ ± 0.68	5.82^Bb^ ± 0.28	6.63^Ab^ ± 0.22	5.98^Ba^ ± 0.45
b* (color anlayses)	First day	19.62^Cb^ ± 0.44	20.05^BCa^ ± 1.38	19.86^Ca^ ± 0.36	20.19^BCc^ ± 0.04	21.13^ABa^ ± 0.16	21.83^Aa^ ± 0.72	21.92^Aa^ ± 0.47	21.46^Aa^ ± 0.55
	Fifth day	20.52^Ba^ ± 0.32	21.66^Aa^ ± 0.16	20.57^Ba^ ± 0.25	20.68^Bb^ ± 0.09	20.76^Bab^ ± 0.07	21.04^ABa^ ± 0.26	21.53^Aa^ ± 0.20	21.26^ABa^ ± 0.97
	Tenth day	18.34^Cc^ ± 0.27	21.24^Aa^ ± 0.48	20.38^ABa^ ± 0.64	21.10^Aa^ ± 0.02	19.31^BCb^ ± 1.51	21.48^Aa^ ± 0.28	21.46^Aa^ ± 0.26	20.30^ABa^ ± 0.87
pH	First Day	7.33^Ba^ ± 0.05	7.53^Aa^ ± 0.05	7.46^Aa^ ± 0.05	7.53^Aa^ ± 0.05	7.46^Aa^ ± 0.05	7.56^Aa^ ± 0.05	7.33^Ba^ ± 0.05	7.30^Ba^ ± 0.10
	Fifth day	7.26^ABa^ ± 0.05	7.30^Ab^ ± 0.00	7.13^BCb^ ± 0.05	7.13^BCb^ ± 0.05	7.16^ABCb^ ± 0.05	7.13^BCb^ ± 0.11	6.96^Dc^ ± 0.05	7.06^CDb^ ± 0.11
	Tenth day	7.30^Aa^ ± 0.00	7.10^Cc^ ± 0.00	7.13^BCb^ ± 0.05	7.20^Bb^ ± 0.00	7.13^BCb^ ± 0.11	7.06^Cb^ ± 0.05	7.20^Bb^ ± 0.00	7.10^Cb^ ± 0.00
Water holding capacity	First day	50.18^CDa^ ± 2.03	51.39^BCb^ ± 1.52	43.14^Eb^ ± 2.07	54.92^Bc^ ± 0.30	53.55^BCb^ ± 0.46	60.45^Aa^ ± 4.17	38.25^Fc^ ± 0.90	47.58^Dc^ ± 1.91
	Fifth day	52.18^DEa^ ± 2.11	55.27^CDa^ ± 1.08	49.23^Ea^ ± 3.25	74.08^Aa^ ± 1.28	65.71^Ba^ ± 2.73	56.47^Cab^ ± 1.93	63.67^Ba^ ± 1.36	65.84^Ba^ ± 0.68
	Tenth day	38.68^Eb^ ± 0.45	49.96^Db^ ± 0.62	29.73^Fc^ ± 0.60	69.81^Ab^ ± 0.16	54.59^Cb^ ± 0.28	53.67^Cb^ ± 0.16	61.69^Bb^ ± 0.21	61.72^Bb^ ± 2.10

*Note:* A–E: Capital letters within a column indicate significant differences (*p* < 0.05) between different yogurt types. a–d: Lowercase letters within a row indicate significant differences (*p* < 0.05) between different storage times. KS, sugar control group; KS5, sugar‐sweetened rice pudding samples supplemented with 5% *Limonium vulgare* Mill. extract; KS10, sugar‐sweetened rice pudding samples supplemented with 10% *Limonium vulgare* Mill. extract; KS15, sugar‐sweetened rice pudding samples supplemented with 15% *Limonium vulgare* Mill. extract; KSE, stevia control group; KSE5, stevia‐sweetened rice pudding samples supplemented with 5% *Limonium vulgare* Mill. extract; KSE10, stevia‐sweetened rice pudding samples supplemented with 10% *Limonium vulgare* Mill. extract; KSE15, stevia‐sweetened rice pudding samples supplemented with 15% *Limonium vulgare* Mill. extract.

* Mean ± standard deviation.

Color is another physicochemical property primarily influenced by various formulations, processing conditions, and storage times. The color parameter was measured using a Hunter instrument, and the lightness (L*) value for the rice pudding samples ranged from 74.41 for KS10 (sample containing sugar and 10% *Limonium vulgare* Mill. extract) to 83.32 for KS (control sugar sample) (Table 1). As the proportion of added *Limonium vulgare* Mill. extract increased in both sugar‐based and stevia‐based samples, a decrease in the L* value was observed. Consequently, darker rice pudding samples were obtained. The observed colorimetric differences among samples can be attributed to variations in the intrinsic pigmentation of raw materials and the synergistic effects of pigments derived from *Limonium vulgare* Mill. extract when interacting with the carbohydrate matrix of sugar and rice flour. Consistent with the findings of Jeske et al. (2017), who reported a whiteness index above 71.3 for commercial plant‐based milks, our results demonstrated a similar trend. Notably, the lowest L* value was recorded in pistachio milk rice pudding, as reported by Ling et al. (2016), with a value of 70.21. The higher L* values observed in cow's milk rice puddings can be attributed to the inherent whiteness of cow's milk, contrasting with plant‐based alternatives. The study by Kadagan and Arslan (2022) reported L* values within a range of 65.71–72.01, demonstrating a decrease in lightness over time. Yeşilyurt (2020) found L* values for rice‐based puddings to be consistent, ranging from 76.81 to 78.06. A comparative study involving various grains and rice pudding as a control indicated L* values of 75.80–75.95 for rice pudding (Köylü 2019). The observed colorimetric differences can be attributed to the varying compositions of the samples, which may have influenced the light reflectance properties. A study examining the potential of nectarines as a sugar substitute in rice and corn starch‐based puddings revealed L* values ranging from 56.24 to 69.20 (Mihaylova et al. 2021). The colorimetric analysis revealed that natural colorants are typically associated with higher L* values (Arocas et al. 2013). The current results corroborate this finding, indicating that the formulations exhibit reduced chroma. Despite the lower chroma, consumers are more likely to perceive these colors as being more natural. Inulin‐enriched dairy desserts, as studied by González‐Tomás et al. (2009), demonstrated higher CIELAB values for all evaluated parameters. Furthermore, other research on starch‐based dairy desserts has reported elevated lightness values (González‐Tomás and Costell 2006). These findings collectively suggest that the color profile of the current formulations deviates from the conventional colorimetric characteristics of puddings.

a* values exhibited significant fluctuations among the rice pudding samples over the storage period. The highest a* value of 7.15 was recorded for sample KS15 (15% *Limonium vulgare* Mill. extract and sugar) on day 5, while the lowest a* value of 2.20 was observed for the control sugar (KS) sample on day 1. The incorporation of both stevia and *Limonium vulgare* Mill. extract resulted in a positive shift in a* values. Conversely, while the control sugar group demonstrated a decreasing trend in a* values, the control stevia group exhibited an increasing trend. The addition of 5% *Limonium vulgare* Mill. extract led to an enhancement in redness, whereas higher concentrations (10% and 15%) resulted in a reduction. Karimidastjerd et al. (2024) determined a* values in the range of −1.5 to −3.6. Most of the rice pudding samples exhibited a generally negative redness value (a*), except for pistachio milk rice pudding, which had a positive a* value. The a* values of rice pudding samples, as determined by Kadagan and Arslan (2022), were found to be between −1.27 and −3.69. The rice pudding samples analyzed in this study displayed positive a* values, a finding directly correlated with the presence of the red pigment from *Limonium vulgare* Mill. The a* values obtained by Mihaylova et al. (2021) were determined to be between 2.88 and 7.62. The positive a* values, attributed to the red pigments derived from apricots, indicated a reddish coloration in the products.

A significant effect of sample type on b* values was observed. The highest b* value of 21.92 was recorded for sample KS15 (15% *Limonium vulgare* Mill. extract and sugar) on Day 1, while the lowest b* value of 18.34 was observed for the control sugar (KSC) sample on Day 1. The control sugar group demonstrated a decreasing trend in b* values over time, whereas the control stevia group exhibited an increasing trend. The addition of *Limonium vulgare* Mill. extract led to a positive shift in b* values. All samples exhibited positive b* values, suggesting a yellowish hue in the overall appearance of the rice puddings. The b* values varied across studies, with Karimidastjerd et al. (2024) reporting a range of 0.1–17.2, Kadagan and Arslan (2022) reporting a range of 4.41–6.63, and Mihaylova et al. (2021) reporting a range of 19.48–26.54. A comparison of our results with those of Mihaylova et al. (2021) revealed a high degree of similarity in terms of b* values. The positive correlation between b* values and yellowness, along with the increased yellow hue observed upon the addition of stevia, supports this finding.

The pH of the samples was found to be within the range of 6.96–7.56. A progressive decrease in pH was observed in all samples throughout the storage period. Samples containing stevia demonstrated higher pH values. The addition of 15% *Limonium vulgare* Mill. extract resulted in a significant decrease in pH, likely due to increased acidity. Moreover, microbial proliferation and the associated increase in acidity during storage further contributed to the observed reduction in pH. Kadagan and Arslan (2022) reported pH values for rice pudding samples within a narrow range of 6.60–6.65, with the control sample exhibiting a slightly higher pH. Similarly, Köylü (2019) found pH values for rice pudding to be in the range of 6.46–6.59. The pH values exhibited significant variability among samples, which could be attributed to the addition of stevia, sugar, and *Limonium vulgare* Mill. extract, as well as the storage conditions.

Water holding capacity data for the rice pudding samples are summarized in Table 1. A progressive increase in water holding capacity was observed over the storage period in samples containing 5% *Limonium vulgare* Mill. and sugar, and 10% *Limonium vulgare* Mill. and stevia, relative to the control sugar and stevia samples. This upward trend was also evident in samples with 5% *Limonium vulgare* Mill. and stevia, 10% *Limonium vulgare* Mill. and sugar, and 15% *Limonium vulgare* Mill. (sugar‐stevia). The water holding capacity values spanned a wide range from 29.73 to 74.08. The results indicate that while 10% *Limonium vulgare* Mill. extract positively influenced water holding capacity, a higher concentration of 15% had a detrimental effect.

Mihaylova et al. (2021) reported water holding capacity (WHC) values ranging from 0.49 to 22.54 in apricot‐added rice pudding. In contrast, our study found significantly higher WHC values, which further increased with storage. Previous research has reported very limited WHC, suggesting an inability to retain nonchemically bound water in the food matrix and subsequent instability under gravity. Some authors have noted that alterations to the original pudding recipe can induce hysteresis loops in aqueous starch pastes (González‐Tomás et al. 2009).

Low WHC can be attributed to the complete substitution of sugar or the inherent lower water absorption capacity of native starches (Liu et al. 2020). WHC is directly correlated with the number of hydrogen bonds formed by starch upon hydration (Deslandes et al. 2019). The limited WHC in previous studies might be due to the lack of extensive temperature damage to starch granules, resulting in fewer available hydroxyl groups for bonding. Maximum swelling of starch granules typically occurs between 70°C and 90°C (Ali et al. 2016). In our study, the addition of stevia likely facilitated increased bonding of hydroxyl groups to starch granules, enhancing swelling. Conversely, direct bonding of hydroxyl groups with sugar might have reduced WHC. However, samples containing stevia exhibited higher WHC, suggesting that stevia may have promoted additional interactions with starch granules, leading to improved water retention.

The sensory analysis results for rice pudding samples are shown in Table 2. Statistical differences were observed among the samples on all storage days. The addition of *Limonium vulgare* Mill. extract led to a decrease in color and appearance scores. The samples supplemented with only stevia exhibited the highest color and appearance scores, whereas the lowest scores were recorded for the samples containing 15% *Limonium vulgare* Mill. extract.

**TABLE 2:** Sensory analyses of rice pudding samples.

Sensory analyses	Storage days	Samples							
		KS	KSE	KS5	KSE5	KS10	KSE10	KS15	KSE15
Color and appearance	First day	4.44^ABa^ *± 1.33	4.55^Aa^ ± 0.72	4.33^ABa^ ± 0.70	4.00^ABCa^ ± 0.50	3.66^ABCa^ ± 0.86	3.44^BCa^ ± 0.88	3.11^Ca^ ± 1.16	3.11^Ca^ ± 1.16
	Fifth day	4.55^ABa^ ± 0.72	4.77^Aa^ ± 0.44	3.66^BCDab^ ± 1.00	4.44^ABCa^ ± 0.72	3.44^Da^ ± 1.01	3.55^CDa^ ± 1.13	3.33^Da^ ± 1.11	3.55^CDa^ ± 0.88
	Tenth day	3.11^Bb^ ± 0.33	3.66^Ab^ ± 0.50	3.00^Bb^ ± 0.70	3.33^ABb^ ± 0.50	2.33^Cb^ ± 0.50	3.44^ABa^ ± 0.72	3.00^Ba^ ± 0.00	3.33^ABa^ ± 0.50
Structure and consistency	First day	3.88^Aa^ ± 0.92	4.00^Aa^ ± 0.70	3.00^Aa^ ± 1.41	3.66^Aa^ ± 1.00	3.00^Aa^ ± 1.22	4.11^Aa^ ± 0.78	3.88^Aa^ ± 1.26	3.88^Aa^ ± 1.26
	Fifth day	3.66^ABa^ ± 1.00	3.88^Aa^ ± 1.05	3.00^ABa^ ± 1.00	3.55^ABa^ ± 0.72	2.77^Ba^ ± 1.20	3.77^ABa^ ± 1.30	4.00^Aa^ ± 0.70	3.55^ABa^ ± 1.01
	Tenth day	2.22^BCb^ ± 1.30	2.66^ABCb^ ± 1.11	2.66^ABCa^ ± 1.22	3.00^ABCa^ ± 1.32	2.11^Ca^ ± 1.05	3.00^ABCa^ ± 1.11	3.33^ABa^ ± 0.70	3.66^Aa^ ± 0.50
Taste and flavor	First day	4.11^Aa^ ± 1.36	4.22^Aab^ ± 0.97	4.33^Aa^ ± 1.00	4.55^Aa^ ± 0.72	3.88^Aa^ ± 0.92	4.44^Aa^ ± 0.52	3.66^Aa^ ± 1.22	4.11^Aa^ ± 1.36
	Fifth day	4.11^ABa^ ± 0.78	4.44^Aa^ ± 0.52	3.44^BCb^ ± 0.72	3.88^ABCab^ ± 0.92	3.00^Cb^ ± 0.70	3.88^ABCab^ ± 1.16	3.33^BCa^ ± 0.86	3.55^BCa^ ± 0.88
	Tenth day	3.11^ABb^ ± 0.33	3.66^Ab^ ± 0.50	3.11^ABb^ ± 0.33	3.55^Ab^ ± 0.52	2.44^Cb^ ± 0.52	3.33^ABb^ ± 0.70	2.88^BCa^ ± 0.78	3.66^Aa^ ± 0.50
General approval	First day	4.44^Aa^ ± 0.88	4.44^Aa^ ± 0.88	4.33^Aa^ ± 0.70	4.33^Aa^ ± 0.70	3.77^Aa^ ± 1.20	4.11^Aa^ ± 0.78	3.55^Aa^ ± 1.23	3.88^Aa^ ± 1.36
	Fifth day	4.11^ABa^ ± 0.60	4.44^Aa^ ± 0.52	3.55^BCab^ ± 0.72	3.88^ABab^ ± 0.60	3.00^Cab^ ± 0.70	3.77^ABCa^ ± 1.09	3.33^BCa^ ± 0.86	3.55^BCa^ ± 0.88
	Tenth day	2.66^ABb^ ± 1.11	3.00^ABb^ ± 1.32	2.77^ABb^ ± 1.09	3.44^Ab^ ± 0.72	2.33^Bb^ ± 0.70	2.88^ABb^ ± 0.78	2.77^ABa^ ± 0.66	3.33^ABa^ ± 0.86

*Note:* A–E: Capital letters within a column indicate significant differences (*p* < 0.05) between different yogurt types. a–d: Lowercase letters within a row indicate significant differences (*p* < 0.05) between different storage times. KS, sugar control group; KS5, sugar‐sweetened rice pudding samples supplemented with 5% *Limonium vulgare* Mill. extract; KS10, sugar‐sweetened rice pudding samples supplemented with 10% *Limonium vulgare* Mill. extract; KS15, sugar‐sweetened rice pudding samples supplemented with 15% *Limonium vulgare* Mill. extract; KSE, stevia control group; KSE5, stevia‐sweetened rice pudding samples supplemented with 5% *Limonium vulgare* Mill. extract; KSE10, stevia‐sweetened rice pudding samples supplemented with 10% *Limonium vulgare* Mill. extract; KSE15, stevia‐sweetened rice pudding samples supplemented with 15% *Limonium vulgare* Mill. extract.

* Mean ± standard deviation.

Sensory analysis revealed that while there were no significant differences in the structure and texture of the rice pudding samples on the first day of storage, these attributes exhibited significant variations on the 5th and 10th days. The incorporation of *Limonium vulgare* Mill. extract consistently resulted in a deterioration of the structure and texture. Similarly, although the taste and aroma profiles were comparable on the initial day of storage, subsequent evaluation days demonstrated significant differences. Notably, the sample fortified with stevia and 5% *Limonium vulgare* Mill. extract achieved the highest sensory scores on the first day, whereas the sample containing sugar and 15% *Limonium vulgare* Mill. extract received the lowest ratings.

Sensory evaluation revealed that while there were no significant differences in overall liking on the initial day of storage, subsequent evaluation days demonstrated significant variations. The samples sweetened exclusively with sugar and stevia garnered the highest overall liking scores on the first and fifth days of storage. However, on the tenth day, the sample containing stevia and 5% *Limonium vulgare* Mill. extract achieved the highest rating. A progressive deterioration in all sensory attributes was evident with increasing storage duration. Despite this general trend, the sample fortified with stevia and 15% *Limonium vulgare* Mill. extract consistently exhibited the highest scores in texture, taste–aroma, and overall liking on the final day of storage. The replacement of sugar with stevia led to improvements in sensory attributes such as color‐appearance, texture, and taste–aroma.

Sensory evaluation indicated that the incorporation of 5% *Limonium vulgare* Mill. extract consistently resulted in higher sensory ratings. Furthermore, in samples sweetened with stevia, the addition of 10%–15% *Limonium vulgare* Mill. extract was found to enhance sensory attributes. Based on the findings of this study, the formulation combining stevia as a sweetener and 10% *Limonium vulgare* Mill. extract yielded a product with superior sensory properties that was highly appealing to consumers.

In another study, the feasibility of using stevia blends as a substitute for traditional sweeteners in high‐protein yogurt was examined. Results indicated that the optimized stevia blend offered a compelling alternative to sucrose and sucralose. Sensory evaluation revealed that the stevia blend exhibited a sensory profile that was highly comparable to that of sucrose and sucralose, while also providing the lowest production cost. The optimized stevia blend, characterized by its pronounced sweetness and lack of off‐flavors, was well‐received by consumers. These findings suggest that the stevia blend has the potential to be a viable substitute for sucrose and sucralose in the formulation of high‐protein yogurt products (Ribeiro et al. 2020).

Despite extensive research on the antimicrobial and antioxidant properties of plant extracts in food products, their application remains limited due to alterations in flavor caused by the high concentrations required for effective antimicrobial activity. While higher concentrations of citrus blossom ( *Citrus aurantium* L.) extract demonstrated significant bactericidal effects, they also led to noticeable changes in sensory characteristics. This study highlights the need for a balance between antimicrobial efficacy and maintaining the product's acceptability. Reducing the extract concentration in the formulation could potentially achieve this balance, ensuring both microbial safety and consumer satisfaction (Degirmenci and Erkurt 2020).

The addition of extracts to functional foods can improve their nutritional value and health benefits. However, increasing the extract concentration can have adverse effects on the sensory qualities of the product, such as taste, aroma, and texture. This can lead to reduced consumer acceptance. Numerous studies have reported a similar trend: as the extract content increases, antioxidant activity is enhanced, but sensory scores tend to decrease.

Total phenolic and antioxidant activity values during the 10‐day storage period are given in Table 3. Phenolic content of rice pudding produced by adding sugar varied between 102.30 and 262.17 mgGAE/g, while phenolic content of rice pudding produced by adding stevia varied between 115.21 and 209.42 mgGAE/g. In the literature, the phenolic content of stevia is reported to vary between 5.76 and 15.05 mg/mL (Sytar et al. 2016). It is thought that the higher phenolic content of stevia rice pudding with up to 5% extract added compared to sugar is due to the phenolic components in the structure of stevia. However, as the rate of extract addition increased, the phenolic substance content of rice puddings to which sugar was added was higher than those to which stevia was added. The reason for this is thought to be the components formed due to the Maillard reaction (Carciochi et al. 2016). The phenolic content of rice puddings produced by adding sugar decreased on the 5th day of storage and increased on the 10th day. This change is thought to be a result of changing pH and the release of complex bound phenolic substance forms due to increased acidity in the later days of storage (Mikulajová et al. 2024).

**TABLE 3:** Antioxidant and total phenolic analyses of rice pudding samples.

Antioxidant and total phenolic analyses	Storage days	Samples							
		KS	KSE	KS5	KSE5	KS10	KSE10	KS15	KSE15
DPPH inhibition (%)	First day	0.40^Dc^ *± 0.27	0.66^Dc^ ± 0.20	4.27^Cc^ ± 0.01	5.10^Cc^ ± 0.98	18.83^Bb^ ± 1.34	18.53^Bb^ ± 0.85	25.75^Ac^ ± 1.73	25.52^Ab^ ± 1.85
	Fifth day	15.70^Ga^ ± 0.19	16.97^Fa^ ± 0.56	20.93^Ea^ ± 1.10	22.38^Da^ ± 0.48	28.89^Ca^ ± 0.90	29.07^Ca^ ± 1.01	35.70^Aa^ ± 0.78	34.26^Ba^ ± 0.04
	Tenth day	3.73^Fb^ ± 0.31	4.63^Fb^ ± 0.27	13.29^Db^ ± 0.27	8.62^Eb^ ± 0.84	19.71^Cb^ ± 1.48	20.20^Cb^ ± 0.90	32.88^Bb^ ± 1.04	35.39^Aa^ ± 0.78
Total phenolic content (mg GAE/g)	First day	116.38^Eb^ ± 1.87	138.30^Db^ ± 6.55	139.43^Db^ ± 6.88	200.12^Ba^ ± 8.57	208.03^Ba^ ± 1.59	163.53^Ca^ ± 1.09	262.17^Aa^ ± 7.57	209.42^Ba^ ± 0.21
	Fifth day	102.30^Ec^ ± 7.21	115.21^Dc^ ± 3.13	119.01^Dc^ ± 5.19	193.51^Aa^ ± 5.67	146.86^Cc^ ± 2.89	167.01^Ba^ ± 2.39	142.75^Cc^ ± 7.19	166.05^Bc^ ± 3.28
	Tenth day	159.33^Ea^ ± 1.70	147.53^Fa^ ± 1.75	163.26^DaA^ ± 5.00	169.56^Cb^ ± 0.59	178.55^Bb^ ± 2.49	165.01^Ca^ ± 3.39	178.64^Bb^ ± 1.52	184.49^Ab^ ± 2.50

*Note:* A–E: Capital letters within a column indicate significant differences (*p* < 0.05) between different yogurt types. a–d: Lowercase letters within a row indicate significant differences (*p* < 0.05) between different storage times. KS, sugar control group; KS5, sugar‐sweetened rice pudding samples supplemented with 5% *Limonium vulgare* Mill. extract; KS10, sugar‐sweetened rice pudding samples supplemented with 10% *Limonium vulgare* Mill. extract; KS15, sugar‐sweetened rice pudding samples supplemented with 15% *Limonium vulgare* Mill. extract; KSE, stevia control group; KSE5, stevia‐sweetened rice pudding samples supplemented with 5% *Limonium vulgare* Mill. extract; KSE10, stevia‐sweetened rice pudding samples supplemented with 10% *Limonium vulgare* Mill. extract; KSE15, stevia‐sweetened rice pudding samples supplemented with 15% *Limonium vulgare* Mill. extract.

* Mean ± standard deviation.

The antioxidant activity value was lower in rice pudding samples containing sugar than in rice pudding samples containing stevia at all storage times. In all samples to which the extract was added, antioxidant activity increased on the 5th day of storage but decreased on the 10th day. It is thought that the phenolic components in the bound form are released due to pH decrease and enzymes on the 5th day of storage, and these components show antioxidant activity. As the storage time increases, it is thought that there is a decrease in the % inhibition value due to the loss of activity of antioxidant components. It has been reported that oxidation and maillard reaction products occurring during storage lose their antioxidant activity at high temperatures (Carciochi et al. 2016). This may have caused sugar‐containing rice puddings to have lower antioxidant activity.

Table 4 presents the total mesophilic, mold, yeast, and coliform counts for the rice pudding samples. The total mesophilic microbial count for the samples ranged from 0 to 3.22 log colony‐forming units (CFU)/g. While an increase in total mesophilic microbial counts was observed over storage time in rice pudding samples containing only sugar, growth was noted on the 10th day of storage in samples containing stevia. The addition of 5% *Limonium vulgare* Mill. extract resulted in the growth of total mesophilic microorganisms, whereas increasing the extract concentration to 10% prevented this growth. The addition of 15% *Limonium vulgare* Mill. extract effectively inhibited microbial growth, particularly in rice pudding samples containing sugar, but caused microbial growth on the final day of storage in the stevia‐containing sample group.

**TABLE 4:** Microbiological analyses of rice pudding samples.

Microbiological analyses	Storage days	Samples							
		KS	KSE	KS5	KSE5	KS10	KSE10	KS15	KSE15
Total number of mesophyll bacteria (log CFU/g)	First day	1.85^Bb^ *± 0.01	0^Db^	0.95^Cb^ ± 0.00	0^Dc^	0^Db^	0^D^	3.22^Aa^ ± 0.03	0^Db^
	Fifth day	2.04^Aa^ ± 0.00	0^Db^	2.03^Ba^ ± 0.00	2.02^Ca^ ± 0.01	0^Db^	0^D^	0^Db^	0^Db^
	Tenth day	2.04^Aa^ ± 0.00	2.04^Aa^ ± 0.00	0^Ec^	1.68^Bb^ ± 0.01	0.61^Da^ ± 0.02	0^E^	0^Eb^	0.91^Ca^ ± 0.03
Number of yeast mold group (log CFU/g)	First day	0^Eb^	2.92^Ba^ ± 0.03	2.95^Aa^ ± 0.00	1.55^Cb^ ± 0.00	0^E^	2.96^Aa^ ± 0.00	0^E^	0.95^Dc^ ± 0.00
	Fifth day	1.69^Aa^ ± 0.00	0^Bb^	0^Bb^	1.69^Aa^ ± 0.00	0^B^	0^Bc^	0^B^	1.69^Aa^ ± 0.00
	Tenth day	0^Cb^	0^Cb^	0^Cb^	0^Cc^	0^C^	1.03^Bb^ ± 0.01	0^C^	1.62^Ab^ ± 0.02
Number of coliform groups (log CFU/g)	First day	0	0	0	0	0	0	0	0
	Fifth day	0	0	0	0	0	0	0	0
	Tenth day	0	0	0	0	0	0	0	0

*Note:* A–E: Capital letters within a column indicate significant differences (*p* < 0.05) between different yogurt types. a–d: Lowercase letters within a row indicate significant differences (*p* < 0.05) between different storage times. KS, sugar control group; KS5, sugar‐sweetened rice pudding samples supplemented with 5% *Limonium vulgare* Mill. extract; KS10, sugar‐sweetened rice pudding samples supplemented with 10% *Limonium vulgare* Mill. extract; KS15, sugar‐sweetened rice pudding samples supplemented with 15% *Limonium vulgare* Mill. extract; KSE, stevia control group; KSE5, stevia‐sweetened rice pudding samples supplemented with 5% *Limonium vulgare* Mill. extract; KSE10, stevia‐sweetened rice pudding samples supplemented with 10% *Limonium vulgare* Mill. extract; KSE15, stevia‐sweetened rice pudding samples supplemented with 15% *Limonium vulgare* Mill. extract.

* Mean ± standard deviation.

Yeast and mold counts in the rice pudding samples ranged from 0 to 2.96 log CFU/g. While yeast and mold growth was observed in samples containing stevia and 10%–15% *Limonium vulgare* Mill. extract, no such growth was detected in samples containing sugar and 5%–10%–15% *Limonium vulgare* Mill. extract. These findings suggest that stevia promoted yeast and mold growth. No coliform bacteria growth was observed in any of the rice pudding samples.

A study involving 100 dairy dessert samples (20 güllaç, 20 kazandibi, 20 keşkül, 20 supangle, and 20 rice pudding) obtained from different markets and pastry shops was conducted. The samples were examined for total mesophilic aerobic bacteria (TMACB), *Staphylococcus aureus* , coliform bacteria, *Bacillus cereus* , yeast and mold, as well as the presence of *Escherichia coli* , *Salmonella* spp., and *Listeria monocytogenes* . The average TMACB count in rice pudding samples was found to be 2.50 log CFU/g, while the average yeast and mold count was 1.18 log CFU/g. Nevertheless, the detection of coliform bacteria at levels below 1.00 log CFU/g indicates that the microbiological quality of these products is generally poor, suggesting inadequate hygienic conditions during production and distribution, and potentially posing a risk to public health (Şahiner et al. 2019). A study conducted by Öksüztepe et al. (2013) on the microbiological quality of dairy desserts consumed in Elazığ revealed an average total mesophilic aerobic bacteria (TMACB) count of 2.30 log CFU/g in rice pudding samples. Likewise, Alişarlı et al. (2002) reported an average TMACB count of 2.16 log CFU/g in rice pudding samples in their study on the microbiological quality of specific dairy desserts. The discrepancy in the results between these two studies may be due to insufficient heat processing during production and/or cross‐contamination resulting from poor hygiene of equipment and personnel post‐heat treatment, or improper storage conditions following production.

Molds and yeasts are allowed in foods only at low levels due to their association with mycotoxin production, infections, and allergic reactions (Aydin et al. 2009). Şahiner et al. (2019) found an average yeast and mold count of 1.18 log CFU/g in rice pudding samples in their study. In a study examining various dairy desserts consumed in the Konya region, Secim and Ucar (2014) reported an average yeast and mold count of 1.03 log CFU/g in rice pudding samples. Similarly, Alişarlı et al. (2002) determined an average yeast and mold count of 0.64 log CFU/g in rice pudding samples in their study on the microbiological quality of certain dairy desserts. In another study, Öksüztepe et al. (2013) reported an average yeast and mold count of 0.39 log CFU/g in 20 rice pudding samples. In our current study, however, the yeast and mold counts were higher than those reported in the literature. These differences in yeast and mold counts may be attributed to inadequate hygiene and sanitation in the production environment, equipment, and storage conditions. Coliform bacteria, considered indicator organisms in foods, provide information about the hygienic conditions and production processes of foods. In the study by Şahiner et al. (2019), coliform bacteria were detected at levels below the detection limit (< 1.00 log CFU/g) in rice pudding samples on average. In a study conducted by Ayok (2002) on rice pudding desserts consumed in Bursa, the average coliform bacteria count was found to be 2.87 log CFU/g, significantly higher than the values obtained in the current study. Secim and Ucar (2014) reported an average coliform bacteria contamination level of 0.66 log CFU/g in rice pudding samples.

Although coliform bacteria were not detected in the examined rice pudding samples, yeast and mold levels were found to be higher than those reported in the literature. This situation indicates that the products may pose significant public health risks during production and storage. As with all foods, it is crucial to implement necessary hygienic measures, Good Manufacturing Practices (GMP), and Hazard Analysis Critical Control Points (HACCP) systems in the production of dairy.

The volatile compound profile of rice pudding samples was presented in Table 5. Major volatile compounds of rice puddings among analyzed samples were benzene, 1,3‐dimethyl, p‐xylene, o‐xylene, gamma‐terpinene, p‐cymene, 2‐nonanone, oxime‐, methoxy‐phenyl‐, vanillin, and 18,18′‐bi‐1,4,7,10,13,16‐hexaoxacyclononadecane.

**TABLE 5:** Volatile compounds of rice pudding samples.

Volatile compounds (%)	Day 1								Day 5								Day 10							
	KS	KSE	KS5	KSE5	KS10	KSE10	KS15	KSE15	KS	KSE	KS5	KSE5	KS10	KSE10	KS15	KSE15	KS	KSE	KS5	KSE5	KS10	KSE10	KS15	KSE15
Ethylbenzene	—	0.73	—	—	—	—	1.75	6.89	—	1.07	—	—	0.79	—	—	—	—	—		0.44	1.19	—	—	—
Benzene, 1,3‐dimethyl—	15.34	11.39	15.79	7.02	10.70	16.36	6.91	—	11.33	32.79	20.47	33.96	18.41	12.68	20.08	17.78	0.93	12.56	60.62	18.98	19.42	17.80	11.64	29.24
p‐Xylene	11.48	26.71	3.25	0.97	4.86	0.88	15.87	4.31	3.59	22.56	—	6.63	1.72	14.57	10.14	12.78	7.56	9.63	7.96	2.96	—	0.10	6.20	—
o‐Xylene	0.70	10.88	4.06	11.76	2.35	1.17	10.60	7.51	9.50	5.26	19.30	23.56	13.55	14.36	12.92	20.75	15.49	2.08	1.40	2.28	11.02	8.78	5.42	0.73
Gamma‐terpinene	2.85	1.83	2.81	4.52	4.36	2.04	6.69	4.28	1.39	1.69	0.96	1.82	2.92	0.67	0.10	5.90	—	1.30	—	6.12	—	3.35	2.47	1.65
Benzene, 4‐ethyl‐1,2dimethyl‐	—	—	—	—	—	—	—	—	—	—	—	—	—	—	—	4.71	—	—	—	—	—	—	—	—
o‐Cymene	—	5.21	—	5.69	—	—	13.49	—	—	4.30	2.99		6.31	—	—	2.64	1.13	—	—	19.97	—	—	0.49	0.47
p‐Cymene	6.34	0.28	5.26	1.17	10.81	5.52	2.24	1.03	8.23	1.44	—	1.74	0.23	—	5.22	3.02	1.00	9.13	—	—	—	8.43	2.62	3.01
Benzene, 1‐methyl‐3‐(1 methylethyl)‐	—	0.07	—	—	—	—	3.89	7.49	—	—	—	2.89	—	0.69	—	—	—	0.68	—	—	—	—	1.79	—
Acetic acid, trichloro‐, methyl ester	3.63	—	0.82	—	2.10	—	1.63	—	—	—	1.69	—	1.80	—	—	—	—	—	—	—	—	—	—	—
2‐Nonanone	—	0.52	0.89	5.40	—	2.82	0.66	1.74	1.45	—	0.33	2.56	0.87	2.47	1.85	—	1.94	1.66	0.75	3.02	2.27	—	0.72	—
2‐Decanone	3.00	—	—	—	—	—	—	—	—	—	—	—	—	—	—	—	—	—	—	—	—	—	—	—
2‐Undecanone	—	—	—	—	—	—	—	—	—	—	—	—	—	0.39		—	—	—	—	—	—	—	—	—
2‐Tridecanone	0.98	—	—	—	—	—	—	—	—	—	—	—	—	—	—	—	—	—	—	—	—	—	—	—
2‐Furanmethanol	—	—	2.60	9.90	0.92	2.94	4.20	14.40	0.42	3.82	—	1.34	3.06	5.94	—	2.53	7.12	0.33	2.47	2.81	7.32	0.96	3.66	0.94
2(3H)‐Furanone, 5‐methyl—	—	—	—	—	—	—	—	—	—	—	—	—	—	—	—	—	—	—	—	—	1.80	—	—	—
Oxime‐, methoxy‐phenyl—	17.39	9.67	10.28	9.06	5.62	3.34	5.16	5.19	4.94	0.71	12.56	2.40	2.89	6.56	8.46	12.74	16.44	9.02	—	3.66	10.56	3.23	7.29	1.68
Hexanoic acid	1.17	—	0.73	—	—	1.89	—	—	—	—	—	1.12	0.53	—	—	—	—	—	—	—	—	1.20	—	3.04
Benzyl alcohol	—	—	—	—	—	—	—	—	—	—	—	0.52	0.44	—	—	—	—	—	—	—	—	—	—	—
Octanoic acid	—	—	—	—	—	—	—	—	0.48	—	—	0.30	—	—	—	—	—	—	—	—	—	—	—	1.58
Oxirane, hexadecyl‐	—	—	—	—	—	—	—	—	—	—	0.50	—	—	—	—	—	—	—	—	—	—	—	—	—
5‐Hydroxymethylfurfural	—	—	—	—	—	—	3.58	—	—	—	—	—	—	—	—	—	—	—	3.71	—	13.24	—	4.21	—
Vanillin	28.72	7.94	17.98	29.89	10.80	29.90	7.43	28.19	9.08	7.95	11.75	18.89	13.80	13.92	5.36	15.77	18.49	14.82	5.56	18.62	15.18	31.01	14.91	37.03
3‐Methylseleno‐2‐benzo[b]thiophenecarboxaldehyde	—	—	0.93	—	—	—	—	—	—	—	—	—	—	—	—	—	—	—	—	—	—	—	—	—
18,18′‐Bi‐1,4,7,10,13,16‐hexaoxacyclononadecane	8.00	15.90	34.62	14.61	37.46	33.02	8.95	18.98	48.79	11.33	29.44	2.29	23.23	21.61	17.13	1.38	29.90	38.80	14.92	21.15	8.39	25.13	38.58	20.63
Benzaldehyde, 3‐hydroxy‐4‐methoxy‐	—	—	—	—	8.66	—	6.76	—	—	6.98	—	—	9.45	6.15	18.74	—	—	—	2.61	—	9.62	—	—	—
Heptaethylene glycol	0.42	—	—	—	—	—	—	—	—	0.05	—	—	—	—	—	—	—	—	—	—	—	—	—	—
3,6,9,12,15‐Pentaoxanonadecan‐1‐ol	—	8.86	—	—	1.35	0.12	0.19	—	0.81	—	—	—	—	—	—	—	—	—	—	—	—	—	—	—

*Note:* KS, control sugar; KS5, sugar and 5% *Limonium vulgare* Mill extract; KS10, sugar and 10% *Limonium vulgare* Mill extract; KS15, sugar and 15% *Limonium vulgare* Mill extract; KSE, control stevia; KSE5, stevia and 5% *Limonium vulgare* Mill extract; KSE10, stevia and 10% *Limonium vulgare* Mill extract; KSE15, stevia and 15% *Limonium vulgare* Mill extract.

Compounds like benzene derivatives and gamma‐terpinene displayed alterations in concentration with the addition of stevia. Specifically, benzene 1,3‐dimethyl‐ was found in greater amounts in samples containing stevia and 5% *Limonium vulgare* Mill. extract compared to those with sugar on Day 10. These results are consistent with research suggesting that stevia influences the composition of aromatic compounds (Gençdağ et al. 2021).

The duration of storage, lasting up to 15 days, had a considerable effect on the volatile profile of the rice pudding samples. Numerous compounds showed significant fluctuations over time, indicating the active stability or breakdown of aroma compounds throughout storage. For instance, the concentration of vanillin increased in certain samples that had higher levels of *Limonium vulgare* Mill. extract, especially in stevia‐based formulations (e.g., KSE15 increased from 28.19% on Day 10 to 37.03% on Day 15). This increase may be attributed to the antioxidant properties of the extract, which could aid in minimizing oxidative degradation during storage (Agregán et al. 2017). In contrast, compounds such as gamma‐terpinene generally decreased in samples, implying potential loss through volatilization or degradation. The storage conditions also influenced the emergence of specific compounds like 2‐furanmethanol and 5‐hydroxymethylfurfural. These compounds, associated with Maillard reactions and thermal sugar degradation, showed a significant tendency to increase over time, particularly in samples kept for 10 days. Such alterations align with earlier studies suggesting that prolonged storage accelerates secondary reactions that contribute to their production (Choudhary et al. 2021).

The inclusion of *Limonium vulgare* Mill. extract notably improved the volatile characteristics of the rice pudding samples by introducing distinctive compounds and amplifying the levels of some existing ones. For example, the concentration of 2‐nonanone, which imparts fruity and floral aromas, typically increased with higher amounts of the extract, particularly in samples using stevia. This supports previous research indicating that plant extracts can enhance the presence of aromatic and phenolic compounds in functional foods (Modupalli et al. 2022). Additionally, the extract contributed to the stabilization of aromatic compounds like vanillin and gamma‐terpinene, likely due to its antioxidant‐rich bioactive components. This stabilization became particularly significant at elevated extract concentrations (e.g., KSE10 and KSE15), where vanillin levels remained high even after prolonged storage. Furthermore, *Limonium vulgare* Mill. introduced unique compounds such as benzaldehyde, 3‐hydroxy‐4‐methoxy‐, which were typically found in samples containing the extract. This compound, related to plant‐derived bioactives, enhances the complexity of the sensory profile and is consistent with research regarding the aroma‐enhancing properties of plant‐based materials (Joung et al. 2016).

The formation of HMF, a well‐known byproduct of the Maillard reaction, is a crucial sign of quality degradation in processed foods and is impacted by variables such as the kind of sugar, storage time, and the presence of polyphenolic compounds (Morales and Jiménez‐Pérez 2001).

According to a number of studies in the literature, sugar‐containing foods exhibit a large rise in HMF generation, particularly when stored at higher temperatures and for longer periods of time (Althaiban 2024). While thermal processing of carbohydrate‐based systems, such as those including sugar, tends to boost HMF formation, alternative sweeteners like stevia may result in significantly lower levels of HMF (Garcia‐Serna et al. 2014). In our study, HMF was not found in the control groups, KS (control sugar), KSE (control stevia), or in certain stevia‐based samples, as shown in Table 6, in line with research findings that indicate less HMF development in systems where stevia is substituted for sugar (Castillo et al. 2013).

**TABLE 6:** Hydroxymethylfurfural (HMF) content of rice pudding samples.

	Storage days	Samples							
		KS	KSE	KS5	KSE5	KS10	KSE10	KS15	KSE15
HMF content (ppm)	1	n.d.^Ba^	n.d.^Ba^	6.09 ± 0.01^Aa^	n.d.^Bb^	n.d.^Ba^	n.d.^Ba^	n.d.^Bc^	n.d.^Bb^
	5	n.d.^Ca^	n.d.^Ca^	n.d.^Cb^	n.d.^Cb^	n.d.^Ca^	n.d.^Ca^	6.16 ± 0.06^Bb^	6.30 ± 0.15^Aa^
	10	n.d.^Ca^	n.d.^Ca^	n.d.^Cb^	6.19 ± 0.13^Ba^	n.d.^Ca^	n.d.^Ca^	6.46 ± 0.23^Aa^	n.d.^Cb^

*Note:* A–E: Capital letters within a column indicate significant differences (*p* < 0.05) between different yogurt types. a–d: Lowercase letters within a row indicate significant differences (*p* < 0.05) between different storage times. KS, control sugar; KS5, sugar and 5% *Limonium vulgare* Mill extract; KS10, sugar and 10% *Limonium vulgare* Mill extract; KS15, sugar and 15% *Limonium vulgare* Mill extract; KSE, control stevia; KSE5, stevia and 5% *Limonium vulgare* Mill extract; KSE10, stevia and 10% *Limonium vulgare* Mill extract; KSE15, stevia and 15% *Limonium vulgare* Mill extract.

Abbreviation: n.d., not detected.

Our study may state that using stevia instead of sugar in rice pudding samples for certain formulations may lessen HMF accumulation.

One important parameter in the production of HMF is storage time. According to the literature, HMF levels significantly rise with storage time (Aslanova et al. 2010). In our study, KSE15 showed higher HMF levels on Day 5, while KS15 and KSE5 samples showed higher HMF levels by the 10th day. The current investigation may support the association between extended storage and HMF accumulation due to the degradation of carbohydrates, which is consistent with the findings of Bulut and Kilic (2009), who found that longer storage times result in greater HMF generation.

The findings of our study indicate that the formation of 5‐hydroxymethylfurfural in rice pudding samples during storage can be effectively inhibited by adding a 10% concentration of *Limonium vulgare* Mill. extract (Table 6). Both sugar (KS10) and stevia (KSE10) containing formulations with 10% *Limonium vulgare* Mill. extract addition showed no HMF formation during storage. However, the samples with lower concentrations (5%) or higher concentrations (15%) revealed noticeable HMF development during storage. According to this finding, it can be underlined that *Limonium vulgare* Mill. extract has a concentration‐dependent inhibitory effect on HMF formation.

The literature has emphasized the efficacy of natural extracts in inhibiting HMF formation in a concentration‐dependent manner. Researches suggest that the effects of these compounds on HMF formation can be both inhibitory and promotive, influenced by their concentration. At lower levels, some antioxidants can significantly lower HMF concentrations by preventing the oxidation of Maillard reaction intermediates. Conversely, when present in higher concentrations, these same compounds may either diminish their protective role or even enhance Maillard reaction products due to their pro‐oxidant characteristics or by promoting the degradation of Maillard intermediates (Oliviero et al. 2009; Jin et al. 2013; Turkut et al. 2018; Abrantes et al. 2022; Hu et al. 2022). In our study, it was observed that when *Limonium vulgare* Mill. extract was used at a 10% concentration, it effectively suppressed HMF formation. However, the same effect was not observed when 5% or 15% concentrations of the extract were used. Similar to findings in related studies, it was determined that the effect of the extract is concentration dependent.

## 4 Conclusion

Sweeteners commonly used in foods are of great importance to both industry and consumers. Therefore, the food industry is striving to offer consumers natural, nutritious, and healthy sweeteners with low‐calorie content. The aim of this study was to reduce the sugar content in rice pudding (a Turkish milk pudding) and to promote the use of *Limonium vulgare* , a plant with high antioxidant activity found naturally in the Amasya region, in various products. It is planned to produce healthier alternative products by the widespread use of stevia, which is newly produced in our country, and *Limonium vulgare* , which is specific to the region, in the industry. By the widespread use of such plants in foods, it is aimed to offer consumers healthy, natural, and nutritious products. This milky dessert produced in this study provides a high nutritional value and calorie‐free functional food for diabetics and children. Stevia use reduces HMF levels and promotes the formation of different aroma components. In addition, *Limonium vulgare* Mill. extract enriches the product in terms of antioxidants and phenolics, while its 15% usage resulted in high scores in terms of sensory evaluation. Moreover, the presence of prebiotic fibers in the stevia plant increases the functional properties of the product. Studies have shown that foods with fiber content have a positive effect on intestinal health. Thus, it has been observed that people who consume fiber‐rich foods have a reduced risk of colon cancer, balanced blood sugar levels, reduced bad cholesterol levels, and improved bone and dental health due to increased calcium absorption. This study will provide both economic support to producers and consumers, and a healthy alternative food will be produced. This study offers consumers a healthy and natural product, while also offering an alternative product to producers. This study provides compelling evidence that substituting traditional sweeteners with stevia and *Limonium vulgare* Mill. extract in sütlaç production can yield a product that is both healthier and more environmentally friendly. The findings underscore the potential of these natural sweeteners to meet the growing consumer demand for cleaner label and sustainable food options.

## Author Contributions

**Fadime Seyrekoğlu:** data curation (equal), formal analysis (equal), investigation (equal), methodology (equal), project administration (equal), software (lead), validation (lead), visualization (lead), writing – original draft (lead), funding acquisition (lead). **Sultan Acun:** data curation (equal), formal analysis (equal), investigation (equal), methodology (equal), visualization (supporting), writing – original draft (supporting). **Aslı Yildirim Vardin:** conceptualization (lead), project administration (equal), resources (lead), supervision (lead), validation (supporting), visualization (supporting), writing – review and editing (lead). **Ali Göncü:** data curation (equal), formal analysis (equal), investigation (equal), visualization (supporting), writing – review and editing (lead).

## Ethics Statement

The authors have nothing to report.

## Conflicts of Interest

The authors declare no conflicts of interest.

## Data Availability

Data will be made available upon request.
